# The economic burden of visceral leishmaniasis and barriers to accessing healthcare in Tigray, North Ethiopia: A field based study

**DOI:** 10.1371/journal.pntd.0012423

**Published:** 2024-10-15

**Authors:** Shewaye Belay Tessema, Tadyos Hagos, Genet Kehasy, Lucy Paintain, Cherinet Adera, Merce Herrero, Margriet den Boer, Haftom Temesgen, Helen Price, Afework Mulugeta

**Affiliations:** 1 Parasitology Unit, Biomedical Department, School of Medicine, College of Health Sciences, Mekelle University, Tigray, Ethiopia; 2 Nursing Department, Ayder Comprehensive Specialized Hospital, College of Health Sciences, Mekelle University, Tigray, Ethiopia; 3 Dignity Period Project, College of Health Sciences, Mekelle University, Tigray, Ethiopia; 4 Department of Disease Control, Faculty of Infectious and Tropical Diseases, London School of Hygiene & Tropical Medicine, London, United Kingdom; 5 KalaCORE Country Office, Addis Ababa, Ethiopia; 6 Sub-Directorate General of Surveillance and Response to Public Health Emergencies, Public Health Agencyof Catalonia, Barcelona, Spain; 7 Médecins Sans Frontières Holland (MSFH/OCA), Amsterdam, The Netherlands; 8 School of Public Health, College of Health Sciences, Mekelle University, Tigray, Ethiopia; 9 School of Life Sciences, Keele University, Keele, United Kingdom; Erasmus MC, University Medical Center Rotterdam, NETHERLANDS, KINGDOM OF THE

## Abstract

**Background:**

Visceral leishmaniasis (VL) is an important public health problem, which mainly affects the poor rural dwelling communities in Low- and Middle-Income Countries. However, little is known about the health and economic burdens of this disease in East Africa, including Ethiopia. The aim of this study was to assess the household level economic burden of VL among affected communities in Tigray, Northern Ethiopia.

**Methods:**

Between April and August 2020, a cross-sectional household survey was conducted on 96 patients who had been treated for VL within 12 months prior to the survey, in six districts of Tigray. Data on households’ health seeking behavior, direct and indirect costs and coping strategies were collected using a structured questionnaire and the responses were analyzed using SPSS software.

**Results:**

Most (82%) of the patients surveyed were males and the majority (74%) of them were between 16 and 30 years of age. The education level of participants was very low: over 33% had not received any form of education; 48% of patients were farmers dependent on subsistence agriculture and about 32% were daily laborers. Just under half of household families (46%) resided in “poor houses” with structures made from entirely local materials. Forty-one percent of patients from the surveyed households had traveled 48 to 72 kilometers to reach VL treatment hospitals. The median total household cost for one VL episode was estimated to be US$ 214. This is equated to 18% of the mean total annual household income or 72.5% of annual per capita income of the study population. More than 80% of the households surveyed incurred catastrophic costs of VL, where this is defined as exceeding 10% of annual household income. The median delay between the onset of symptoms and arrival at a care provider hospital was 37 days; once the patient arrived at hospital, the median delay during diagnosis was 3 days. Direct and indirect costs represented 44% and 56% of the total costs incurred, respectively. To cope with VL treatment costs, 43% of the households used more than one coping strategy: 48% took out loans, 43% sold livestock and 31% of households mobilized cash savings.

**Conclusions:**

VL in Tigray is concentrated among young males with low educational background and mostly engaged in subsistence economic activities. Despite the free diagnostic and treatment provisions that were available at public hospitals at the time of the study, our work shows that the household economic burden of the disease had significant impact among VL-affected communities in Tigray. Initiating community awareness towards prevention, early treatment seeking and decentralization of VL treatment centers are strongly recommended. In addition, we recommend efforts to reduce household treatment costs through transport and food provisions for patients (and their accompanying carers where possible) or through cash reimbursement for patients who complete treatment at public hospitals, in order to reduce the barriers to seeking treatment for this life-threatening disease.

## Author summary

Visceral leishmaniasis (VL) is a vector-born parasitic disease which predominantly affect poor communities in isolated regions and is closely linked with extreme poverty. VL is life-threatening when left untreated and has important socio-economic impacts. In this study we examined the economic burden of VL among households in Tigray, northern Ethiopia. Our findings indicate that the economic burden of VL as a percent of household income is extremely expensive, with a large proportion representing catastrophic health costs. The economic impact of VL is not only limited to high cost of treatment, but also time lost during treatment-seeking periods and hospital stays. VL diagnostics and drugs are being provided free of charge to patients treated at public hospitals, an important policy measure in Ethiopia. Nevertheless, the economic impact of the disease is still very challenging to households. Therefore, VL transmission needs to be blocked by implementing efficient prevention strategies, mainly through outreach case-detections and community awareness campaigns to alleviate the economic burden of households from VL endemic communities. In the absence of effective prevention, the other feasible way is through decentralization of VL care centers to enable patients get care services nearby without spending much time and transportation costs.

## Introduction

Leishmaniases comprise a complex of neglected vector-borne parasitic diseases, caused by more than 20 species of obligate, intracellular protozoan parasites of the genus *Leishmania*; mainly transmitted through a bite of infected female Phlebotomine sandflies [1]. The leishmaniases group of diseases have diverse clinical patterns: ranging from self-healing localized skin ulcers, cutaneous leishmaniasis (CL), metastasizing mucocutaneous leishmaniasis (MCL) and diffuse cutaneous leishmaniasis (DCL) to the lethal systemic manifestation of visceral leishmaniasis (VL, also known as kala azar) [2].

VL predominantly affects poor communities [3] in isolated regions and is closely linked with extreme poverty, economic migration, inadequate medical care and decreased immunity (through malnutrition) [4]. A decade ago, Eastern Africa was next to South Asia in the global VL burden; however, in a recent (WHO 2022) global report, East Africa (Eritrea, Ethiopia, Kenya, Somalia, South Sudan, Sudan and Uganda), was found to be the most VL-affected region in the world, shouldering over 66% of the global VL cases [5] reported. In the Horn of Africa, VL often occurs with devastating epidemics and its burden is mainly concentrated in three countries: in Sudan with an estimated incidences of 15,700 to 30,300 cases per annum (p.a); in South Sudan 7,400 to 14,200 cases p.a. and in Ethiopia 3,700 to 7,400 cases p.a. [6].

Understanding the socio-economic impacts of VL and documenting the economic burden of the disease on affected populations would help to inform policy makers and concerned bodies on their decision-making, in order to devise and implement feasible approaches for disease prevention and control programs. In this regard, the household economic burden of VL has been much more widely studied and considered in South Asian VL-endemic countries [4,7,8] compared to East African countries.

VL mainly affects the productive (15–45 years old) age group of the rural communities in Ethiopia and outbreaks often occur during harvesting seasons [9]. The disease is considered as a major health burden in Tigray and Amhara regions of northwest Ethiopia, but in a country characterized by limited resources and multiple competing health issues, the rationale for public investments that address VL is strengthened if it can be shown that the disease represents a significant economic burden to the affected communities. However, information on the full economic impacts of the disease in Ethiopia and in the Horn of Africa in general is incomplete. Therefore, this study was carried out to estimate the economic burden of VL among households in Tigray, Northern Ethiopia.

## Material and methods

### Ethical considerations

Ethical clearance (Ref: ERC 1396/2019) was obtained from the Research Ethics committee and Institutional Review Board (IRB) of the College of Health Sciences of Mekelle University and a support letter (Ref: 882/1418/11) was granted by the Tigray Health Bureau (THB). Patient’s medical records were reviewed retrospectively, and all information retrieved from medical records was anonymized. Information about the study was provided to potential participants and verbal informed consent was obtained from all adult patients or representatives of their household, and from a parent or guardian of participating minors during door-to-door /household visits/ interviews.

### Study setting

Tigray is located in the Northern part of Ethiopia between 12° 15‘N and 14° 57‘N latitude and 36° 27‘E and 39° 59’E longitude. Tigray shares borders with the Afar Region to the east, Eritrea to the north and north-east, Sudan to the west and Amhara Region to the south and south-west. The Tigray region is administratively divided into 7 Zones and 93 Woredas. The 93 woredas are further divided into 813 sub-districts (locally called Tabias) [10]. Tabias are the smallest administrative units in the region. The landscape of Tigray is characterized by lowland plains and high plateau mountains. The western and northwestern lowland zones of the region bordering with Gedarif state of Sudan and Eritrea are known for a high prevalence of VL.

### The health care system and VL case management in Tigray

The public health care system in Tigray is organized across a three-tiered delivery structure. These consist of the primary health care unit, PHCU (health posts, health centers and primary hospitals), secondary care (general hospitals) and tertiary care (specialized hospitals). The catchment population of a PHCU is estimated to be 60,000 to 100,000 people, a secondary care area serves 1.0 to 1.5 million people, and a tertiary care institution serves approximately 3.5 to 5 million people. In Tigray, six hospitals provide inpatient and outpatient health care services for VL patients in the region. These are Kahsay Abera Hospital (Humera) and Meareg Hospital (Dansha) of Western zone, St Mary Hospital (Aksum) from the Central zone, Sihul Hospital (Shire) and Maeini Hospital (Shiraro) of Northwestern zone and Ayder Comprehensive Specialized Hospital situated in Mekelle City. However, relatively very few VL cases were treated at Meareg and Maeini Hospitals. VL cases are detected using passive methods through patients who seek medical services at public health care centers. The most common diagnostic method for VL is an immunological rapid diagnostic dipstick test (RDT) for detection of rK39 antibodies. When patients with suspected VL test negative for rK39 response, microscopic analysis is performed using Giemsa-stained spleen aspirates (SA) or bone marrow aspirates (BMA) [11,12]. All patients with a confirmed diagnosis of VL are admitted (tertiary level care) for the full duration of treatment to ensure 100% adherence and to monitor drug side effects. The first-line VL treatment drug in Tigray and Ethiopia in general is a combination of sodium stibogluconate (SSG) and paromomycin. The sodium stibogluconate (20mg/kg body weight/day) and paromomycin (15mg/kg body weight/day) combination therapy is administered intramuscularly for 17 days [13] in the public hospitals of the region.

### Study design

A cross-sectional household survey was undertaken between April and August 2020 in six districts (locally Woredas) of Tigray Regional State, Northern Ethiopia. The direct and indirect costs of a VL episode from the household perspective are presented. The provider perspective is not included.

### Study population

The study population was all VL patients (or caregivers from the household) who sought treatment at Sihul-Shire and St Mary–Axum hospitals.

### Sample size and sampling technique

In total, 96 VL patients (or care takers) were recruited and included in the study. Prior to the study site selection, over 5 years (2015–2019) annual report data documented at the Tigray Regional Health Bureau (TRHB) was reviewed to identify the number of patients treated for VL and to determine these patients’ geographic locations. In the TRHB annual reports document, over the five years, the majority of VL treated patients were from western and northwestern zones, followed by the central zone of Tigray. Kahsay Abera, St Mary and Sihul-Shire hospitals had the highest VL caseloads. However, Kahsay Abera hospital was excluded for security reasons. Ayder, Meareg and Maeini Hospitals had a relatively small VL case load; hence, they were excluded from this study. Therefore, considering operational feasibility in terms of security reasons, logistic resources and the likely numbers of VL patient admissions per facility, Sihul-Shire and St Mary public hospitals were purposively selected for this study.

**Fig 1:**
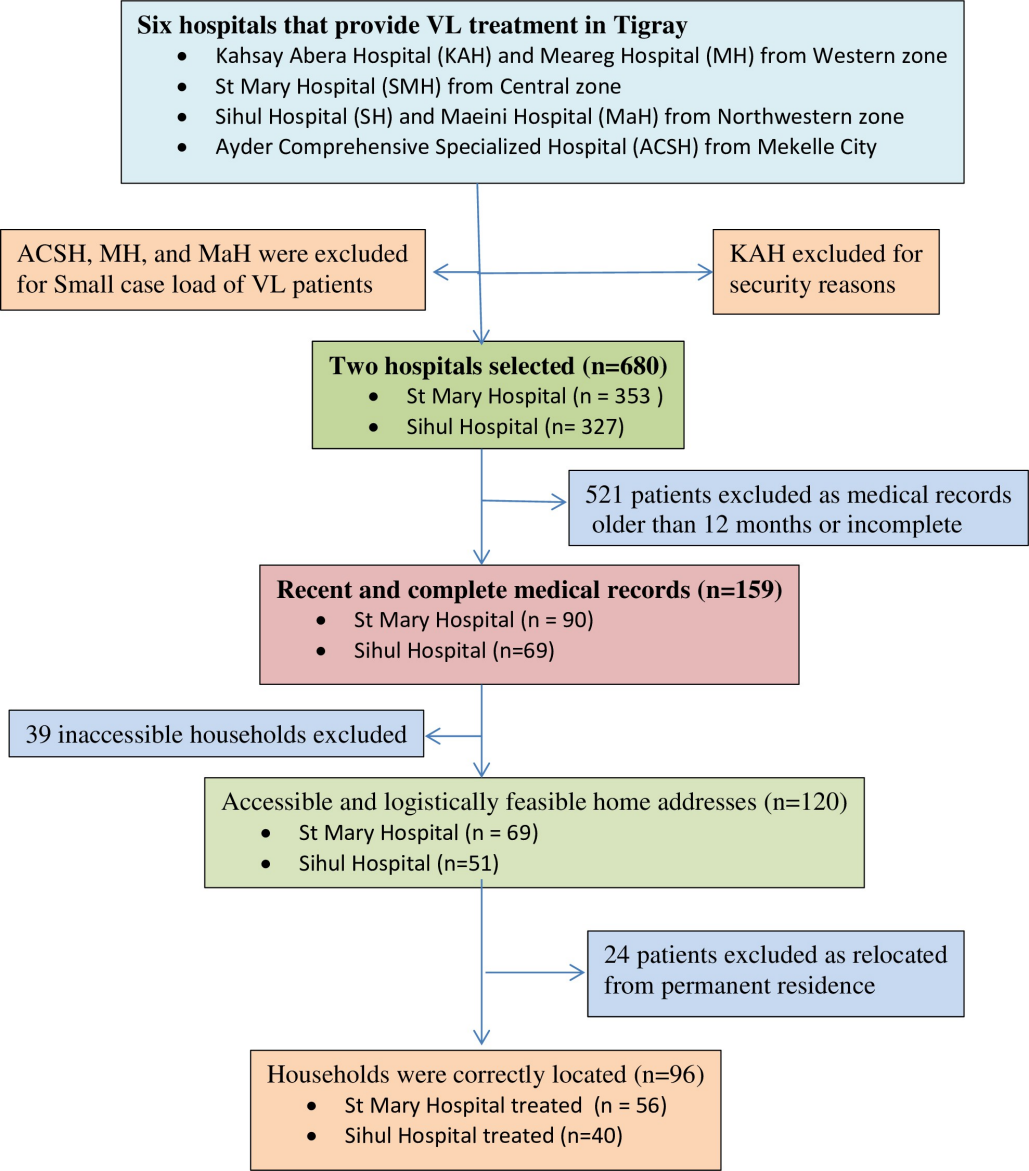
Schematic representation of the sampling procedure used to select the study sites and study participants (n = 96).

Study households were determined using VL patients’ hospital records. Individual respondents were recruited and then interviewed in their place of residence through house-to-house visits. Over the previous five year period (2015 to 2019) prior to data collection, a total of 680 people were reported as VL treated patients in the selected two hospitals, 353 at St Mary-Axum and 327 at Sihul-Shire hospitals. Medical records of the VL patients treated at the two hospitals were reviewed to determine the treatment periods of these individuals. To minimize recall bias, patients who were treated within the last 12 months (Sept 2018 to Aug 2019) prior to the data collection period were included. Using the hospital medical records at both hospitals, a total of 159 patients (90 from St Mary and 69 from Sihul hospitals) were identified as having had VL treatment during the last 12 months prior to the date of data collection. All 159 patient treatment charts were reviewed to determine the accessibility of each patient home address for household survey through door-to-door interviews and 120 patients were found with accessible and logistically feasible home addresses in their medical charts. However, 24 households with a treated case of VL were not found at the locations indicated by the hospital notes. The remaining 96 households were correctly located at the specified patient addresses and invited to take part in the study (Fig 1).

### Study variables

The main study variables of this study were socio-demographic characteristics of VL patients and their households, household income, household assets, health-seeking behavior, direct treatment expenditures (medical and non-medical costs), indirect treatment costs (loss of productive time values) and household coping strategies used during the most recent VL episode.

### Data collection methods

Data were collected using a pre-tested structured questionnaire administrated by a team of trained and experienced senior health professional data collectors who had previously been involved in VL related household surveys. In the absence of the patient at the time of interview, the head of the household or any responsible adult caregiver above 18 years selected by the family as being the most knowledgeable person of the household was approached. Prior to the household visits, medical records of the most recent VL patients treated at St Mary and Sihul hospitals were reviewed for the type of diagnosis, treatments given and treatment duration (hospital stays) of patients. The study participants were interviewed in their resident backyards.

### Measurements

#### Income sources of households

To estimate the median monthly household income, the survey team collected data on the economic activities of all household members. For patients and caregivers who stated farming as their main source of income, the survey collected data on the household annual production, which was then valued with local market prices and divided by the number of agriculturally active household members. For household members who reported daily or casual labor as their main source of income, the wage rate was determined by asking a series of questions on the daily or monthly cash revenues. We also estimated total household income as the sum of monthly cash income from daily labor for each economically active household member and income from agricultural products.

#### Direct cost

Data on direct medical and non-medical costs were collected from households (service users) incurred for each care provider they had visited. These included all out-of-pocket expenditures by the household during the most recent single VL episode. Direct medical costs included registration, consultation, laboratory examinations, drug purchases and other medical supplies at each provider. Direct non-medical costs included expenditures on transportation to and from the health care provider, food costs incurred while travelling to a health care provider and during hospitalization at the treatment facility and other daily out-of-pocket expenditures for the patient and accompanying caregiver(s).

#### Indirect cost

The indirect cost of a VL episode represented the loss of productive time for the VL patient and his/her caregiver(s) and was estimated using the human capital approach. The loss of productivity was valued in terms of the loss of earnings of the patient and household members caring for the patient (either at home or hospital). Patients and their caregivers were asked about the number of days they were unable to engage in productive activities, and this was multiplied by a median daily income to obtain the indirect cost of a VL episode. For patients and caregivers who reported daily labor as the main source of income, the daily wage rate was estimated and multiplied by the number of working days lost to obtain the indirect cost of the VL episode. Here, the loss of work days among housewives (engaged in unpaid work activities at home) and the loss of school attendance days of student patients/caregivers were excluded when estimating the loss of income to household. Because, the loss of work days in both (housewives and students) cannot be converted into a monetary value. Besides, those who depended on monthly wages or government employed salaried household members who secured leave certificate from their employers during the VL episode were excluded when estimating the loss to household since there is no reduction of monthly salary income due to the loss of productive days. However, all patients were considered when estimating total economic loss at household level.

#### Data analysis

The collected data were entered into EPI Info version 20.1.14 statistical package and exported to SPSS Statistical package version 23. Due to skewed distribution, data patterns in the income and health cost lists, results were mainly summarized by medians and quartiles. All costs were assessed in Ethiopian local currency (ET Birr.) and converted to United States Dollars (USD) considering the average annual value of currency exchange rate prevailing at the Central Bank of Ethiopia in 2019 (1 USD = ET Birr 28.91).

#### Catastrophic costs

To obtain the median total household cost, the costs incurred by households on the most recent VL episode including both direct (medical and non-medical out-of-pocket expenditure) and indirect costs (productive time losses due to illness) were added together. Then, the median total cost outcome was divided by the annual household income. According to the budget share approach, the costs of VL were considered catastrophic if they exceeded 10% of the annual household income [7,14,15,16].

## Results

### Characteristics of study participants

In this study, a total of 96 VL patients (56 treated at St Mary and 40 treated at Sihul-Shire hospital) participated, all of whom were permanent residents in the Tigray region. A majority of the study participants (82.3%, 79/96) were males. The median age of the patients was 23 years (IQR = 20–30 years) and the majority, 74% were between 16 and 30 years of age, followed by 13.5% of patients who were within the age range of 31–45 years. The education level of our study participants was very low; more than 33% had not received any form of education and about 67% of patients had received some formal education. From those who had formal education (n = 64), over 56% had attended primary school (1–6 grades), about 42% had secondary level (7–10 grades) education and only one individual had completed technical/vocational (10+3) school, which was the highest education level among all study participants. Subsistence agricultural practices were the main sources of income for 48% of patients (n = 46). Most households (78.1%, n = 75) owned livestock, either cattle, sheep, goats, donkey, camel and/or chickens with a median headcount of 1–5 animals (Table 1).

**Table 1:** Socio-demographic characteristics of study patients and household assets (n = 96).

Variable	Number	Percent (%)
**Gender**		
Males	79	82.3
Females	17	17.7
**Age (years)**		
1–15	4	4.2
16–30	71	74.0
31–45	13	13.5
>45	8	8.3
Median family size	4	
**Educational background**		
Unable to read and write	32	33.3
Primary (1–6 grades) school	36	37.6
Secondary (7–10 grades) school	27	28.1
Technical/ vocational (10+2/3) school	1	1.0
**Occupation**		
Farmer	46	47.9
Daily laborer	31	32.3
Civil servant	1	1.0
Student/ Child	13	13.6
Housewife	5	5.2
**Household assets**		
Television	8	8.3
Radio	25	26.0
Mobile cellphones	46	47.9
Bicycle	3	3.1
Animal-drawn cart	2	2.0
**Access to electric power**		
Yes	25	26.0
No	71	74.0
**Main source of fuel for cooking meal**		
Firewood	82	85.4
Charcoal	12	12.5
No cooking in dwelling	2	2.1
**Animal owning**		
Yes	75	78.1
No	21	21.9
**Type of owned animal (median headcount)**		
Cattle (5)	75	78.1
Goat (10)	49	51.0
Sheep (5)	19	19.8
Chicken (5)	68	70.8
Camel (1)	8	8.3
Donkey (1)	38	39.6
**Housing status**		
Poor ^a^	44	46
Moderate ^b^	36	37
Good ^c^	16	17

^a^ Mud walls with grass roof or wall and roof made of stone, wood and soil (without cement)

^b^ Mud-plastered walls with corrugated iron roof or waterproof tiles

^c^ Cement plastered walls and waterproof tiles.

### Effects of VL on ability to work

Over 80% (n = 78) of the participants were economically active at the time their illness occurred. During the most recent VL episode, 95% of the patients (n = 91) were unable to perform their full-time daily activities due to illness; of those, 30% (n = 29) were able to perform daily activities sometimes and 65% (n = 62) were unable to perform any work at all.

### Health care seeking and access to VL treatment services

A primary health care unit (health post, health center or a primary hospital) was the first choice for the majority of households (52%, n = 50), followed by a general hospital as first point of contact for 18% of households. Before presentation to qualified health providers at the VL treatment hospitals, patients had visited a median of three other low level health care providers (IQR 2–3.8). In this study, more than 40% of the patients had travelled between 48–72 kilometers (km) to reach the VL treatment hospitals at either St Mary or Sihul hospitals. While traveling to the VL treatment centers, the majority (80%) of patients had used public transport (bus), 8.3% patients traveled on foot, 6.2% used private transport (bajaj & motorbike) and 5.2% used a public ambulance. The median delay between the onset of symptoms and the patient reaching the VL treatment hospital (i.e., the patient delay before VL diagnosis and treatment) was 37 days (IQR 19–67). Lack of awareness on VL (44.8%), delayed referral (34.4%) and financial problems (20.8%) were the main reasons for the delays in seeking care. Once the patient had presented to a qualified provider at the VL treatment hospital, the median delay to correct diagnosis was 3 days (IQR 2–7). Proximity to home was the main reason given for the choice of the first care provider for 63% of households (n = 60) (Table 2).

**Table 2:** Health care seeking behaviour of VL patients (n = 96).

Variable	Number	Percent (%)
**Type of health care provider first visited**		
Traditional healer	7	7.3
Private Drug shop / Pharmacy	10	10.4
Primary health care unit	50	52.1
Hospital (General or Referral)	17	17.7
Private clinics	12	12.5
**Main reason to choose first care provider**		
Proximity to home	60	62.5
Perceived (good) reputation	29	30.2
Other reasons	7	7.3
**Number of health care providers visited**		
1	2	2.1
2	43	44.8
3	34	35.4
4	12	12.5
5	5	5.2
Number of care providers visited, median (IQR)	3 (IQR 2–3.8)	
**Delay to VL treatment hospital presentation in days, median (IQR)**	37 (IQR 19–67)	
**Main reason for delay to visit VL treatment hospital**		
Due to patient’s lack of awareness about VL	43	44.8
Delay referral due to lower knowledge of the care providers	33	34.4
Due to financial constraints	20	20.8
**Delay to diagnosis at VL treatment hospital in days (median; IQR)**	**3 (IQR 2–7)**	
**Means of travel to visit VL treatment hospital**		
Foot	8	8.3
Private vehicle (Bajaj & motorbike)	6	6.2
Public transport (Bus)	77	80.3
Ambulance	5	5.2
**Distance between home and VL treatment facility (kilometers)**		
<24	0	0
24–48	33	34.4
48–72	39	40.6
72–96	20	20.8
>96	4	4.2
**Number of caregivers accompanying patient**		
0	6	6.3
1	79	82.3
2	7	7.2
3	4	4.2
Number caregivers accompanying patient (median: IQR)	1 (IQR 1–2)	

### Diagnosis and treatment of VL

**Fig 2:**
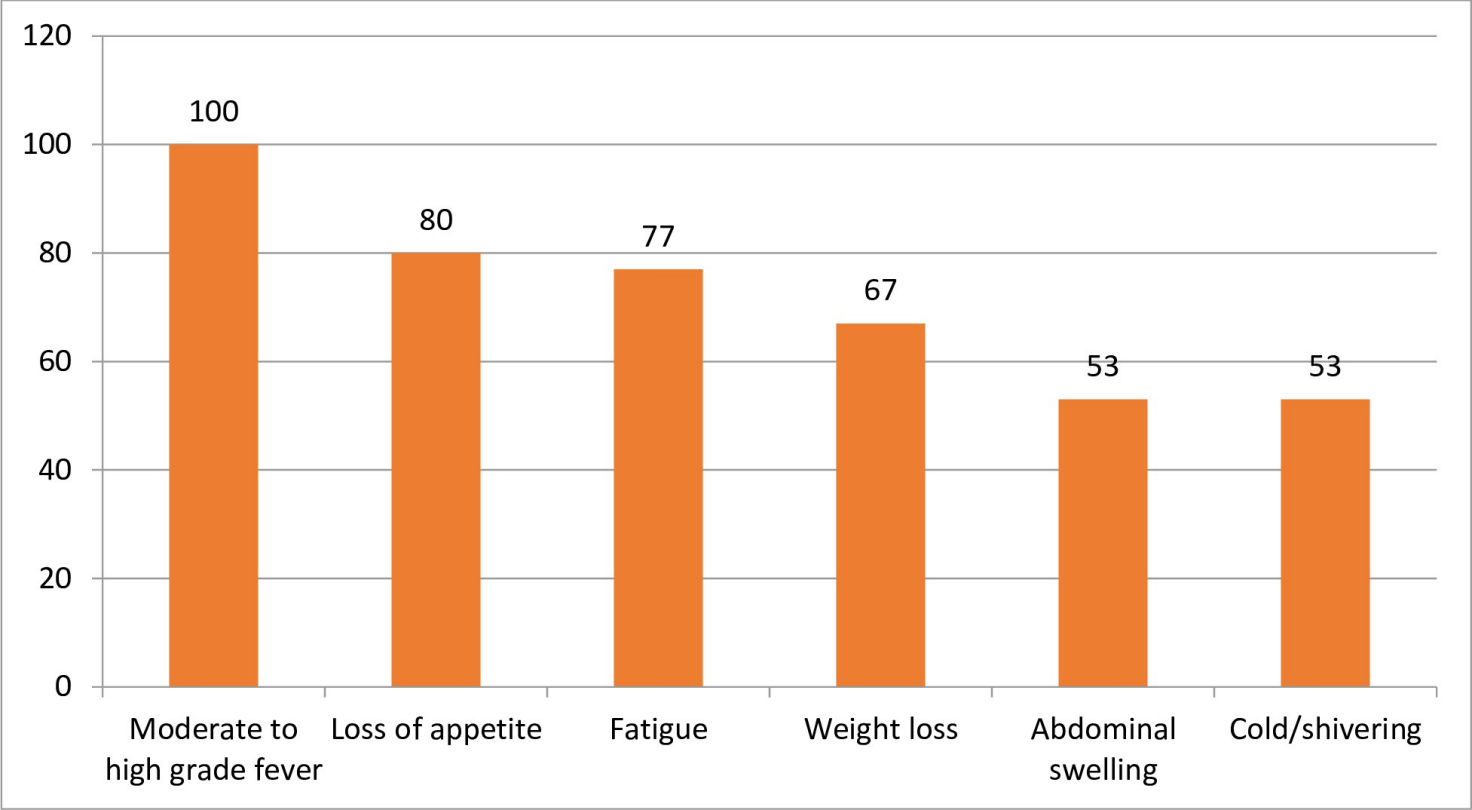
Main symptoms of VL patients (shown as %) in St Mary and Sihul General Hospitals (n = 96).

VL is usually characterized by one or more debilitating symptoms, many of which are shared with other infectious diseases such as malaria. Among the main symptoms of VL recorded in the patients’ charts were moderate to high grade fever (100%), loss of appetite (80%), fatigue (77%), weight loss (67%), abdominal swelling (53%) and cold or shivering (53%) (Fig 2). rK39 dipstick for blood specimen and microscopy for spleen and bone marrow aspirates were the two common diagnostic methods used in 75% (n = 72) and 25% (n = 24) of the VL patients, respectively. Similarly, 68.8% (n = 66) were treated using paromomycin and sodium stibogluconate (PM-SSG) combination therapy given intramuscularly for 17 days; and 26% (n = 25) of them were treated with AmBisome (liposomal amphotericin B) administered 5mg/kg/day over a period of 6 days (i.e. 30mg/kg in total). The remaining patients (5.2%, n = 5) were treated with SSG monotherapy; administered as intramuscular injection of 20mg/kg/day for 30 days. The median hospital stay of VL patients as inpatients at the VL treatment hospitals was 19 days (IQR 14–36).

### Direct costs

The households incurred a median total direct cost of US$ 101 across all providers visited during the most recent VL incident (IQR US$ 67–145); where about 63% of the total direct cost (US$ 64; IQR US$ 46–93) was non-medical cost and about 30% of the total direct cost (US$ 30; IQR US$ 18–48) was medical expenditure. The total direct costs were mainly used for food, ancillary drugs, laboratory diagnosis, accommodation, transportation to/from hospital and other expenses. Relative to the costs at public institutions, the median direct medical costs were higher for households visiting private care providers (e.g. pharmacies and clinics). Households visiting private pharmacies spent a median cost of US$ 25 (IQR US$ 17–39) mainly for auxiliary drugs purchase only (Table 3).

**Table 3:** Direct medical and non-medical costs to VL patients from various health care service providers in Tigray (in USD, 2019).

Direct costs	Traditional healer (n = 31)		Drug shop or Pharmacy (n = 12)		Private doctor or Clinic (n = 60)		Primary health care unit (n = 94)		VL treatment General Hospitals (n = 96)		Total costs of all provider	
	Mean (Sd)	Median (IQR)	Mean (Sd)	Median (IQR)	Mean (Sd)	Median (IQR)	Mean (Sd)	Median (IQR)	Mean (Sd)	Median (IQR)	Mean (Sd)	Median (IQR)
Direct medical costs												
Consultation a	-	-	-	-	3(1)	2(2–3)	1(0)	1(0–1)	2(3)	1(0–3)	4(4)	3(2–5)
Laboratory diagnosis	-	-	-	-	11(8)	10(4–17)	2(2)	1(0–2)	5(4)	4(2–7)	13(9)	11(5–20)
Treatment ^b^	6(6)	4(3–5)	27(12)	25(17–39)	6(7)	4(3–5)	7(15)	4(0–7)	3(4)	1(0–4)	19(21)	12(6–23)
Total direct *medical* costs	6(6)	4(3–5)	27(12)	25(17–39)	19(11)	16(10–26)	9(15)	7(3–11)	10(6)	8(5–13)	36(26)	30(18–48)
Direct *non–medical* costs												
Transport ^c^	6(5)	5(3–8)	8(7)	8(2–13)	4(4)	2(1–6)	2(8)	0(0–2)	5(6)	3(1–9)	13(12)	10(4–17)
Food cost ^d^	14(9)	17(2–17)	5(4)	6(3–8)	9(8)	11(0–17)	11(13)	8(0–17)	14(5)	14(10–17)	36(20)	32(21–47)
Accommodation ^e^	5(6)	4(0–7)	4(4)	5(0–9)	3(4)	0(0–7)	4|(5)	2(0–9)	6(6)	5(0–10)	14(11)	12(7–21)
Other	1(1)	1(0–1)	5(5)	3(0–10)	2(4)	1(0–3)	2(5)	0(0–1)	6(7)	4(0–10)	10(10)	8(1–14)
Total direct *non-medical* cost	25(12)	29(16–32)	23(13)	22(12–33)	18(13)	19(6–29)	20(17)	17(7–28)	32(9)	32(26–37)	74(38)	64(46–93)
Total direct cost	31(14)	34(19 = 42)	49(19)	51(39–64)	38(16)	39(29–47)	29(24)	25(14–38)	42(8)	41(36–46)	110(55)	101(67–145)

a Include payments for entry registration and medical file; b Includes costs for ancillary drugs, syringe and accommodation at provider institutions; c Includes travel cost of caregiver; d The total food cost since patients left their home including their caregivers; e Accommodation cost including their caregiver.

### Indirect costs

The median number of workdays lost by a patient was 58 days (IQR 40–100) and the median loss of income for working patients was US$ 71 (IQR US$ 47–118) compared to the median loss of income across all working and non-working patients estimated to be 65 US$ (IQR US$ 45–115). Moreover, a VL patient was attended/accompanied by a median of 1 caregiver (IQR 1–2). These working caregivers had lost a median of 22 workdays (IQR 14–36) and income of 42 US$ (IQR US$ 25–66) (Table 4).

**Table 4:** Duration of illness, number of caregivers, lost workdays, loss of income and indirect costs per VL episode in US$ (1US$ = 28.91 ET Birr in 2019).

Variables	Number of days			
	Mean	(sd)	Median	(IQR 25–75)
Patients’ duration of illness (days)^a^	70	44	58	40–100
Missed school days of student patients (n = 13)	22	19	20	18–38
Number of caregivers per VL patient	1.3	0.6	1.1	1.0–2.0
Lost workdays of working caregivers (n = 86)	25	13	22	14–36
Missed schooldays of student caregivers (19)	26	14	22	14–36
Indirect costs of household	Estimated costs in US$			
	Mean	(sd)	Median	(IQR 25–75)
All patients (n = 96)	80	52	65	45–115
Working patients only (n = 78)	86.8	52.3	70.6	47–118
All caregivers (n = 105)	25	18	22	14–40
Working caregivers only (n = 86)	45	32	42	25–66
*Total indirect cost to household*	132	58	120	90–161

^a^ Includes different delays (on treatment seeking and delays during diagnosis) and patients admission stays at treatment facilities.

### Household income

The median monthly income of an economically active or working patient and income of the households (working and non-working) was estimated to be US$ 34.6 (IQR US$ 26–51) and US$ 98 (IQR US$ 56–150.4), respectively. Similarly, the median annual household and per capita income was US$ 1,180 (IQR US$ 675–1,804) and US$ 295 (IQR US$ 169–451), respectively (Table 5).

**Table 5:** Summary of total household income and total costs (direct and indirect) incurred per single VL episode (1US$ = 28.91 ET Birr in 2019).

Household income and treatment costs		Amount in American Dollar (US$)			
Variables		Mean	(sd)	Median	(IQR 25–75)
Annual income:					
	• Household income	1390	1125	1180	675–1804
	• Per capita income	348	281	295	169–451
Direct and indirect costs per VL episode:					
	• Direct medical cost	36	26	30	18–48
	• Direct non-medical costs	74	38	64	46–93
	• Indirect costs	132	58	120	90–161
	*Total household costs*	236	180	214	122–343
Median total costs (direct and indirect) as a % of:					
	Annual household income	18.1%			
	Annual per capita income	72.5%			
Catastrophic health cost = Direct + indirect costs / annual households’ income = 94+120/1180		18.1%			
	Proportion of households exceeding 10% threshold = 77/96	80.2%			

### Total costs to a household due to a single VL episode

The median total household cost for one VL episode was found to be US$ 214 (Table 5). The median direct expenditure by households, including the health-seeking phase and the costs incurred at the treatment facilities was US$ 101 (IQR US$ 67–145), whereas the median total lost time value (i.e. indirect cost) to the household per episode of VL was US$ 120 (IQR US$ 90–161). The median annual household income was estimated to be US$1,180 (IQR US$ 675–1,804) and the median annual per capita income was US$ 295 (IQR US$ 169–451).

The economic burden of VL to households, including direct and indirect costs, was equal to 18.1% of the median annual household income or 72.5% of the median annual per capita income of the study population.

Health cost was catastrophic for 80.2% of households in this study, meaning that for these households direct out-of-pocket expenditure (both medical and non-medical) and indirect costs exceeded 10% of annual household income.

### Household coping strategies

Households used four main strategies to cope with financial expenses associated with VL treatments, namely: borrowing money, remittances from family and friends, selling household assets such as livestock, and using their savings (Table 6).

**Table 6:** Household coping strategies and costs in US$ (1US$ = 28.91 ET Birr in 2019).

Coping strategy	Households		Amount of money in US$			
	Number	Percent (%)	Mean	SD	Median	IQR
Sold livestock animals (n = 75)	32	42.7	73	42	78	41–86
Mobilizing Savings (n = 96)	30	31.3	138	72	138	69–208
Loan (n = 96)	46	47.9	73	47	69	23–138
Remittances (n = 96)	41	42.7	55	45	14	7–55

## Discussion

This is the first published study to provide information on the cost estimates and economic burden of VL (kala azar) in Tigray, and Ethiopia in general. We present a comprehensive set of evidence quantifying the estimated treatment cost to a household per single VL episode, the economic burden of the disease and common financial coping strategies used by affected households.

The median total direct and indirect costs per single episode of VL was estimated to be US$ 214 (IQR US$ 122–343). The VL health care related household expenditures in Tigray did not include costs for diagnosis and drug treatment for the disease, because they are provided free of charge at public hospitals. Otherwise, the median household cost of a single treatment would escalate further from the current figure of US$ 214 to approximately US$ 277 assuming the estimated VL drug cost using WHO recommended prices of US$ 63 per patient [17]. Health care cost is defined as catastrophic health when it exceeds 10% of the household annual income [7]; or when the costs of VL health care services caused households to divert money from supporting basic needs or to use their savings planned for other purposes [3,18,19]. In this study, the economic burden of VL (as a % of household income) was 18.1% of the annual household income or 72.5% of median annual per capita income and more than 80% of households exceeded the 10% catastrophic cost threshold. Our findings were higher compared to the 51% of patient households exceeding the catastrophic cost threshold and treatment costs of 57% of the median per capita income from Nepal [7]. In another study carried out in Sudan, using both care provider and service user perspectives, the economic burden of VL was 40% of the annual household income and over 83% of households were shown to exceed the 10% catastrophic cost threshold [15].

The amount of direct and indirect costs households incurred were 44% and 56% of total costs, respectively (Table 5). This was in line with a study report from Nepal, where the direct and indirect costs were represented 47% and 53% of total costs, respectively [7]. Households incurred a median total direct cost of US$ 101 (IQR US$ 67–145). This was lower than the direct cost reported from Sudan where households incurred US$ 185 [15] per single patient treatment. However, the current result was higher than the direct costs reported from Nepal where households incurred a total median direct cost of US$ 66 per single VL episode [7]. Similarly, about 63% of the costs (US$ 64; IQR US$ 46–93) were non-medical expenditures. A study from Sudan identified food cost for patients and caretakers as the leading direct non-medical cost of 85% incurred during VL treatment periods, in line with our study findings [15]. The higher expenditures on food were perhaps born from long duration hospital stays of patients and caregiver(s) at VL treatment hospitals suggesting the need for cash reimbursement program as practiced in Brazil, Nepal and some Asian countries [7,20,21].

In the present study, about 95% of the patients were unable to carry out their daily activities. The current study showed that median of 58 productive days lost among patients in Tigray was lower than the workdays lost in Bihar, India, where 120 productive days were lost among working patients [22]. The figure found in this study was slightly higher than the 51 working days lost among working patients and caregivers in Sudan [15] and 57 days lost among patients and caregivers in Nepal [7]. Although the loss of days allocated for school attendance cannot be converted into a monetary value, the disease had a severe impact on household members’ primary or secondary school attendance which is likely to cause an indirect economic loss to affected households in Tigray. The large proportion of farmer patients in this study was comparable to the report from Ethiopia where VL mainly affected the rural farming communities [9]. The economic impact of VL in Tigray is not only limited to high cost of treatment, but also time lost during treatment-seeking periods and hospital stays during treatment.

Delay to access healthcare at the VL treatment hospitals was common. Lack of awareness, delays in obtaining a referral and financial constraints were the main reasons for delayed treatment of people with VL (Table 2). In partial agreement with our study findings, a study report from Nepal has indicated that many patients delay seeking healthcare due to a lack of awareness about the disease and its severe consequences, confusing symptoms of the disease with other conditions, together with fear of productivity losses [23,24]. The most common reason acknowledged by households (44.8%) was lack of knowledge on VL. The exposure to media sources increases with increasing education. Educated patients can access the mainstream and social media regularly and such exposure to these media increases awareness on VL and other diseases. Educated patients are more likely to read and listen to various media outlets. In agreement with our study findings, the patients with lower education in Sudan had poor knowledge on VL [15]. There is no community awareness creation activity towards prevention and control of the disease in the study area. Besides, the care provider personnel serving at primary care facilities do not routinely receive training on VL and there is fast turnover of the experienced care providers. Therefore, the care providers serving at primary care facilities may have limited knowledge about the disease and less likely to be able to give counseling and early referrals for patients who consulted them. Therefore, the longer patient delay before arrivals at the treatment hospitals observed in this study was probably in part due to low patient awareness about VL and its consequences when delayed for treatment. Besides, the other possible reasons for the longer patient delay to get treatment at the VL care provider hospitals were partly due to low awareness of care providers at primary care facilities and partly due to the long distances needed to travel to the limited VL treatment hospitals from patients’ homes.

Almost half (48%) of the households who hosted VL patients were headed by poor farmers depending on subsistence agricultural practices and residing in “poor houses”, with non-permanent structures (wall and roof) made from entirely natural materials, mud-plastered wall grass roof huts or houses with semi-permanent structures made of stones, wood and soil. In agreement with our findings, about 59% of people with VL were poor individuals dwelling in mud floor and tin wall houses in Bangladesh [25]. Various studies have revealed that VL is a disease of poverty [26,27]; primarily affecting the poorest communities in the isolated regions of the globe [3,28,29,30,31]. Therefore, homes with low-quality housing structures (walls and roofs with cracks or holes) in Tigray might be serving as suitable sites for the insect vectors (sandflies) to reside and breed within them. Houses with low-quality structures may aggravate the probability of anthroponotic disease transmission through indoor transmission. Therefore, community awareness creation activities towards indoor infection prevention through sealing cracks and holes in walls and roofs are among the crucial steps needed to be taken [32,33].

In the current study, taking out loans, receiving remittances, selling livestock and mobilizing cash/savings were the main strategies used to cope with the higher treatment costs of VL in Tigray. In partial agreement with our study findings, taking out loans (often with high-interest rates), receiving remittances from families or friends, use of cash deposit and sale of household properties were among the reported coping strategies commonly used by poor household families elsewhere [23,24,26]. However, for the poor subsistence farming communities, animals are the most important household assets. Animals and animal products are the main sources of food and the main tools for their agricultural activities and crop production. People cannot plow their plot of land without animals, especially without oxen. When these oxen are sold to cover VL treatment expenditures, the households become unable to cultivate their lands. They will be forced to rent or to sell their plot of land and become unable to farm and this reinforces poverty in the household. The use of cash savings for VL treatment out of their predetermined plans added further poverty to households. Thus, this study result has indicated that despite drugs being provided free of charge, the direct and indirect costs of VL care services were extremely expensive to the patients and their household families relative to their income, with a large proportion of VL incidents representing a catastrophic health expenditure.

### Strength and limitations of the study

This is the first published study to estimate the household economic burden incurred per single VL episode on treatments in Tigray and Ethiopia at large. This study also had limitations. First, this was a household survey with participant recruitment based on two VL treatment hospitals only which may limit the generalizability. The study patients were selected through passive surveillance from cases detected and treated at two public care provider hospitals (St. Mary and Sihul hospitals). Therefore, the actual VL costs in other care providers including private hospitals in the Tigray region may be underestimated. Secondly, treatment cost data from care providers (hospitals) perspective was not included. We have used treatment costs from the service users (households) perspective only. Therefore, the actual economic burden of the disease from a full societal perspective is underestimated. Third, due to security problems in western Tigray and for logistic reasons, only households located in geographically reachable and logistically feasible districts (Woredas) were included in this study. However, patients located in very remote and hard-to reach areas could incur more treatment costs; perhaps, having a significant increase in the economic burden of VL to households. Therefore, the current result of VL economic burden up on households could be undervalued in this study. Fourth, we only analyzed the most recent single VL episode per household. In this study, the last single VL episode per household was analyzed and relapse cases were not included. Obviously, repeated treatment cost expenditures would significantly increase the economic burden of the disease to the household. Therefore, we likely underestimated the true economic burden of VL upon the households in Tigray. Last but not least, patient recall bias was the other limitation for this study. Due to the long delay (one year), patients may face difficulties in recalling exact costs for VL treatment.

## Conclusions and recommendations

Despite VL diagnostics and drugs being provided free of charge to VL patients, this study has indicated catastrophic health cost in over 80% of households affected by this life-threatening disease. In order to reduce the financial barriers and improve access to VL diagnosis and care, the free VL diagnosis and drugs at public health facilities in Tigray and in northern Ethiopia at large, have been an important policy measure. Without this policy, the economic burden of VL would have been much higher. Nevertheless, the economic impact of VL is still very challenging for households and needs great attention due to substantial direct and indirect costs to patients and their households. Therefore, intensified efforts from all concerned bodies (governments, NGOs and the international community) are needed to reduce the economic burden of VL among disease affected communities in Tigray. To reduce the household economic burden of VL, disease transmission needs to be blocked by implementing efficient prevention and control strategy through outreach case-detections and community awareness campaigns. In the absence of effective prevention and control measures, the other feasible way to alleviate the disease burden is through decentralization of VL care centers as a means to reduce the number of providers visited, reduce delays of accessing VL treatment and enabled patients to get care services easily without much time and transport costs. In order to alleviate the household treatment cost, introducing cash reimbursement program for VL treated patients would help to cover the wider range of treatment costs being incurred by households and may improve healthcare seeking behaviors and reduce delays in accessing VL treatment.

### Special remark

The study was carried out before the devastating war waged on Tigray in November 2020. Prior to the war, Tigray had been able to create one of the relatively better health systems in Ethiopia as evidenced by the rapid expansion of access to health services in the region. However, the war in Tigray erupted in November 2020 and brought unimaginable humanitarian crisis and enormous damage to the health system in the region [34]. As a result, the health system in Tigray almost collapsed; about 81% of the village health posts, 74% of the health centers, 80% of the primary hospitals and 86% of the hospitals (secondary and tertiary) were fully or partially damaged and/or vandalized/looted and hence the majority of the health facilities went non-functional. Those few in operation have been functioning at a much lower capacity due to the displacement, relocation, migration, injury and/or death of the health workforce and shortage of drugs, medical supplies and equipment. Worst of all, while VL is fatal when left untreated, there was no VL care service anywhere in Tigray during the last two years. Currently, although VL care service has restarted recently in Ayder, St Mary and Sihul-Shire hospitals, three of the hospitals are suffering from lack of medical equipment, insufficient drug supplies and above all due to scarcity of trained health staffs. Thus, the war has predisposed the entire population of Tigray to devastating untimely deaths, irreversible disability, mental and physical illnesses from communicable diseases (including VL) and non-communicable illnesses. Furthermore, the war caused a massive internal displacement of the civilian population into the surrounding bushes and forests (*Acacia* and *Balanites)* for fear of the heavy artillery bombardments, human rights abuses and civilian casualties. The bushes and forest canopies (*Balanite aegptica* and *Acacia* trees) could have been helpful at hiding the people; but at same time they are the fertile breeding sites for the vector (sand fly) and reservoir hosts thereby transmitting VL parasites and increasing the risk of infections. Regrettably, the war induced internal displacement to the bushes and forests together with the absence of health care services is believed to have alarmingly increased the health and economic burden of VL in these war-affected communities of Tigray, even further beyond the high levels reported by this study.

## Supporting information

S1 Table A summary of household income and indirect cost of VL episode to households; monthly income, annual income and Per capita income in ETH Birr and US Dollar. (DOCX)

S2 Table A summary loss of income among productive patients, productive caregivers and a median total indirect cost to households per single VL episode. (DOCX)

S1 Text Catastrophic Cost estimation and calculation methods. (DOCX)
