# Supplementary material for: The economic burden of visceral leishmaniasis and barriers to accessing healthcare in Tigray, North Ethiopia: A field based study
Source: PLoS Negl Trop Dis. 2024 Oct 15;18(10):e0012423. doi: 10.1371/journal.pntd.0012423 (PMC11508124; doi:10.1371/journal.pntd.0012423)
Supplement: S1 Table — (DOCX) [file pntd.0012423.s001.docx]

*S1 Table.* A summary of household income and indirect cost of VL episode to households; monthly income, annual income and Per capita income in ETH Birr and US Dollar.

| Productive HH members (n=78) | | Individual Monthly income | | HH Monthly income | | Monthly Per capita | | Household Annul income | | Annual  Per capita | |
| --- | --- | --- | --- | --- | --- | --- | --- | --- | --- | --- | --- |
| Currency Type | | Birr | US$ | Birr | US$ | Birr | US$ | Birr | US$ | Birr | US$ |
| Median income | | 1000 | 34.6 | 3250 | 112.5 | 813 | 28.1 | 39000 | 1349.0 | 9750 | 337.3 |
| IQR | 25% | 750 | 25.9 | 2438 | 84.3 | 609 | 21.1 | 29250 | 1011.8 | 7313 | 252.9 |
|  | 50% | 1000 | 34.6 | 3250 | 112.5 | 813 | 28.1 | 39000 | 1349.0 | 9750 | 337.3 |
|  | 75% | 1462.5 | 50.6 | 4753 | 164.5 | 1188 | 41.1 | 57038 | 1972.9 | 14259 | 493.2 |
| All productive & non-productive HH members (n=96) | |  |  |  |  |  |  |  |  |  |  |
| Median income | | 875 | 30.3 | 2843.8 | 98.4 | 710.9 | 24.6 | 34125.0 | 1180.4 | 8531.3 | 295.1 |
| IQR | 25% | 500 | 17.3 | 1625.0 | 56.2 | 406.3 | 14.1 | 19500.0 | 674.5 | 4875.0 | 168.6 |
|  | 50% | 875 | 30.3 | 2843.8 | 98.4 | 710.9 | 24.6 | 34125.0 | 1180.4 | 8531.3 | 295.1 |
|  | 75% | 1337.5 | 46.3 | 4346.9 | 150.4 | 1086.7 | 37.6 | 52162.5 | 1804.3 | 13040.6 | 451.1 |

IQR= Inter quartile range; HH= household; ETH = Ethiopian; Birr = Ethiopian currency
