# Supplementary material for: The economic burden of visceral leishmaniasis and barriers to accessing healthcare in Tigray, North Ethiopia: A field based study
Source: PLoS Negl Trop Dis. 2024 Oct 15;18(10):e0012423. doi: 10.1371/journal.pntd.0012423 (PMC11508124; doi:10.1371/journal.pntd.0012423)
Supplement: S2 Table — (DOCX) [file pntd.0012423.s002.docx]

S2 Table. A summary loss of income among productive patients, productive caregivers and a median total indirect cost to households per single VL episode

| HH members | | Patients (Productive n = 78) | | | Caregivers (Productive n=86) | | | Patients + Caregivers | |
| --- | --- | --- | --- | --- | --- | --- | --- | --- | --- |
| Out of work & Income loss | | Out of work | Income loss amount | | Out of work | Income loss amount | | Total loss of income to households | |
| Measurement | | Days | Birr | US$ | Days | Birr | US$ | Birr | US$ |
| Median | | 58 | 2040 | 70.6 | 22 | 1207 | 41.8 | 3481 | 120.4 |
| IQR | 25% | 40 | 1370 | 47.4 | 14 | 578 | 20.0 | 2595 | 89.8 |
|  | 50% | 58 | 2040 | 70.6 | 22 | 1207 | 41.8 | 3481 | 120.4 |
|  | 75% | 100 | 3420 | 118.3 | 40 | 1759 | 60.9 | 4653 | 160.9 |

IQR= Inter quartile range; HH= household; ETH = Ethiopian; Birr = Ethiopian currency
