# Supplementary material for: The economic burden of visceral leishmaniasis and barriers to accessing healthcare in Tigray, North Ethiopia: A field based study
Source: PLoS Negl Trop Dis. 2024 Oct 15;18(10):e0012423. doi: 10.1371/journal.pntd.0012423 (PMC11508124; doi:10.1371/journal.pntd.0012423)
Supplement: S1 Text — (DOCX) [file pntd.0012423.s003.docx]

S1 Text. Catastrophic Cost estimation and calculation methods:

A budget share approach at 10% threshold [14, 15,16] was used to calculate the catastrophic costs. First, the direct (medical and non-medical) costs and indirect costs of a household were added together to obtain the median total cost of a single VL episode. Then, to obtain the catastrophic cost estimate, the total household cost was divided by the annual household income.

Median total household income and treatment costs estimated:

- Annual household income= 1180
- Annual per-capita income =295
- Total direct medical costs of HHs =30
- Total direct non-medical costs of HHs=64
- Median total indirect cost of HHs = 120
- Total HH treatment cost per single VL Episode=214

Catastrophic cost = Direct cost (medical + non-medical cost) + Indirect cost/ annual household income

The median total cost as a percentage of annual household income = (30 +64)+120/ 1180 = 18.1%

The median cost as a percentage of annual per-capita income =214/295.1 =72.5%
